# Depressive disorders: Treatment failures and poor prognosis over the last 50 years

**DOI:** 10.1002/prp2.472

**Published:** 2019-05-03

**Authors:** Thomas P. Blackburn

**Affiliations:** ^1^ TPBioVentures Hoboken New Jersey USA

**Keywords:** antidepressants, clinical trials, depression, diagnosis, ketamine, monoamines, SSRIs

## Abstract

Depression like many diseases is pleiotropic but unlike cancer and Alzheimer's disease for example, is still largely stigmatized and falls into the dark shadows of human illness. The failure of depression to be in the spotlight for successful treatment options is inherent in the complexity of the disease(s), flawed clinical diagnosis, overgeneralization of the illness, inadequate and biased clinical trial design, restrictive and biased inclusion/exclusion criteria, lack of approved/robust biomarkers, expensive imaging technology along with few advances in neurobiological hypotheses in decades. Clinical trial studies summitted to the regulatory agencies (FDA/EMA) for approval, have continually failed to show significant differences between active and placebo. For decades, we have acknowledged this failure, despite vigorous debated by all stakeholders to provide adequate answers to this escalating problem, with only a few new antidepressants approved in the last 20 years with equivocal efficacy, little improvement in side effects or onset of efficacy. It is also clear that funding and initiatives for mental illness lags far behind other life‐treating diseases. Thus, it is no surprise we have not achieved much success in the last 50 years in treating depression, but we are accountable for the many failures and suboptimal treatment. This review will therefore critically address where we have failed and how future advances in medical science offers a glimmer of light for the patient and aid our future understanding of the neurobiology and pathophysiology of the disease, enabling transformative therapies for the treatment of depressive disorders.

**Abbreviations**
CDS: Carroll Depression Scale
ECT: electroconvulsive therapy
EMA: European Medicines Agency
GWAS: genome‐wide association
LOCF: Last Observation Carried Forward

## 1 INTRODUCTION

In his book “Better than Prozac, Creating the Next Generation of Psychiatric Drugs,” Dr Samuel Bardones, argued “that many antidepressants fail miserably because of the many flaws. Even the best of them are blunt instruments that have a large number of effects on the brain, only some of which are considered therapeutic. The fact we understand the reason for their limitations and what must be learned before we can expect substantially better antidepressants,”^1^ Some would argue that such therapeutic limitations would suggest that we are still decades away from achieving this goal. Be that as it may, a recent publication in the Lancet, entitled “Comparative efficacy and acceptability of 21 antidepressant drugs for the acute treatment of adults with major depressive disorders: a systemic review and network meta‐analysis,” would both challenge this pronouncement and suggest we already have effective antidepressants.^2^ This recent study and several others purport that SSRI's do work in depression (but only perhaps in subtypes? ‐ see later discussion) and that some older second‐generation antidepressants (eg, amitriptyline) showed greater efficacy, than many SSRI's.^2^, ^3^ Thus, questioning the need for the next generation of “better” antidepressants and the need to step forward from historical dogma, redundant clinical classification, to a new era of neurobiology, neuronal networks of depression and precision pharmacology with its focussed on diagnosis based science not symptoms.^4^, ^5^, ^6^ Given, the high failure rate of antidepressant clinical trials the proposition for better antidepressants remains in question, given that in the real‐world antidepressants are only efficacious in 30%‐40% of depressed patients and probably in subpopulation yet to be identified^7^. Thus, patients with mental disorders deserve better treatments and a move away from symptom‐based diagnosis is urgently needed. A move in the right direction with a shift to biologically based diagnosis was initiated in 2008 by the NIMH as part of a long‐term strategic initiative with their Research Domain Criteria Project (RDoc), Insel, 2014. The success of this and other biologically based initiatives are now emerging and potentially will be game‐changing and discussed in more detail later in this review.

As a neuropsychopharmacologist, I've spent a lifetime in the preclinical discovery and clinical development searching for “better” antidepressants with an improved efficacy, side effect profile and importantly a rapid onset of action. Thereby, continually building on what we have learned over 50 years of research, with some limited success and failures along the way (*The author has directed preclinical and clinical programs for several antidepressant drugs. Viloxazine Vivalan* ^*®*^ *, paroxetine, Seroxat* ^*®*^ */Paxil* ^*®*^ *, SmithKline Beecham's selective 5‐HT*_*2A&,B&C*_, *5‐HT6 receptor antagonists, SNAP37889/HT2157 (Galanin‐R3 antagonist), SNAP7941 (MCH*_*1*_ *antagonist) and trace amines‐like compounds*.

The Holy Grail has always been to find a novel antidepressant with (a) improvements in the efficacy; (b) speed of onset; (c) safety/tolerability and (d) a reduction in remission rates and relapse/recurrence; (e) without severe withdrawal syndrome, to alleviate the symptoms of this debilitating mental disorder and life‐threatening illness. The challenge I faced like many neuropharmacologists and clinicians, is the immense complexity of the disease, its causes, difficulties in diagnosis and the failure of numerous clinical studies—so common in testing new drugs for neuropsychiatric disorders and for that matter in other therapeutic areas.

Depressive disorders, in particular major depression disorder (MDD) is based on a 50‐year‐old monoamine hypothesis, questionable animal models and subjective clinical diagnostic criteria, with comorbidity across several neuropsychiatric disorders.^8^, ^9^

Thus, the focus of this review is to ask the question(s) again, why are there so many failures and why so few successes and do we need “better” antidepressants? In this review, I want to build on what we've learned from the past and how this may lead us to future clinical successes in the treatment of depressive disorders. First, we need to understand the complexity this neuropsychiatric disorder, the global crisis, unmet medical needs, its current diagnosis, treatment, and future areas of research.

## 2 GLOBAL DEPRESSION CRISIS—THE UNMET MEDICAL NEED FOR BETTER ANTIDEPRESSANTS?

Depression is a significant contributor to the global burden of disease and affects people in all communities across the world requiring better treatment options for patients. The World Mental Health Survey conducted in 17 high income countries found that on average about 1 in 20 people reported having an episode of depression in the previous year. Depression is the leading cause of disability worldwide in terms of total years lost due to disability. The demand for curbing depression and other mental health conditions is on the rise globally. Antidepressant use has increased from 7.7% in 1999‐2002 to 12.7% in 2011‐2014 both sexes and 16.5% for females (see links below).

A recent World Health Assembly called on the World Health Organization and its member states to take action in this direction.

https://www.cdc.gov/nchs/data/databriefs/db283_table.pd#4).

(http://www.who.int/mental_health/management/depression/wfmh_paper_depression_wmhd_2012.pdf).

It is estimated that the prevalence of depression in the US is 15% percent of the population reportedly taking an antidepressant—if not more. MDD is ranked fourth as a disease measured in disability adjusted for life years (DALYS) in 1990.^7^, ^10^ Together with the fact that available antidepressant medications are ranked second behind ischemic heart disease as a potential disease burden by 2020. The risk for MDD, especially for females in developed countries, is 1 in 10. And, there is considerable evidence that depression is associated with increased risk for cardiovascular and infectious diseases as well as immunological and endocrine changes. The World Health Organization predicted that depression will become the leading cause of human disability by 2020.^11^ It has been estimated that over a lifetime, the global prevalence of depression is 21.7% for females and 12.7% for males who suffer from depression at some point.

Epidemiological studies have estimated that 5%‐9% of women and 2%‐3% of men in the US suffer from depression at any time.^8^ And, a Norwegian study showed that 24% of women suffer major depression at some point in their lives and 13.3% suffer from dysthymia, while 10% of males suffer from major depression at some point, and 6% suffer from dysthymia.^8^ Depression in children and adolescents is a cause of substantial morbidity and mortality in this population, being a common disorder that affects 2% of children and up to 6% of adolescents^12^, ^13^. Although antidepressants are frequently used in the treatment of this disorder, there has been major controversy about the efficacy and safety of these medications in this population.^7^ This led to the US food and Drug Administration (FDA) publishing a list of recommendations from the Psychopharmacologic Drugs and Paediatric Advisory Committees over the years, including many other neurological and psychotropic drugs.

This critical appraisal on the treatment of depression in children and adolescents is still an area of great concern and controversy in relation to the developing brain. Depression is a common condition with up to 8% of all teenagers having met criteria for depression in the last year.^14^ In fact, by the age of 21 years, up to 14.8% of individuals have met criteria for a mood disorder.^13^, ^15^

Some types of depression are familial, indicating that there is inherited vulnerability.^16^ Similarly, in studies of families in which members of each generation develop bipolar affective disorder (BPAD) it has been found that those with the illness have a different genotype from those who do not become ill. Conversely, the reverse is not true: not all individuals with a purported BPAD genotype will develop the illness (epistasis—mutations in one gene masks a phenotype at another locus). That in additional to other factors, stresses at home, work, or school or other coping skills, are involved in the onset of the disease. In some families, major depression also seems cooccur generation after generation.^17^

A National Institutes of Mental Health (NIMH) National Comorbidity Survey of more than 9000 US adults in 2005; using the Diagnostic and Statistical Manuel of Mental Disorders—DSM‐IV‐TR (Text Revision 2001) criteria, found that 6% of those studied had a debilitating mental illness, yet treatment was difficult to obtain, with only one‐third or more of those in care receiving minimally adequate treatment, such as appropriate drugs or a few hours of therapy over a period of several months. In general, the investigators found that things had not changed much over the past decades and would argue that the situation has deteriorated further in recent years. In an earlier Massachusetts Institute of technology (MIT) survey the estimated direct and indirect cost of mood disorders in the US to be $43 billion in 1990. In a more recent MIT survey (including a wider range of disorders and costs, plus EU member countries), estimated the total in 2010 to be $780b, of which 60% was attributable to direct costs and 40% to lost productivity.^18^, ^19^ Depressed individuals incur twice the medical cost burden as nondepressed patients, the main part (80%) being for medical care rather than psychiatric or psychological services, with the bulk of antidepressant prescriptions (80% worldwide) being written by primary care physicians. (PCP's). With up to 30% or more of patients with MDD who do not respond to typical antidepressant medications.^20^

Alternative effective treatments for moderate‐to‐severe depression include a combination of somatic therapies (CDT, pharmaco‐therapy, repetitive transcranial magnetic stimulation (rTMS), transcranial direct‐current stimulation (tDCS), and the more established electroconvulsive therapy (ECT). ECT has been rejuvenated for the treatment for the most severe, melancholic depressions, particularly in the elderly (who are more prone to adverse effects of drugs) and in approximately 30% of patients who do not respond to SSRI antidepressants (treatment resistant patients—see later for further discussion). However, patient accesses to alternative treatments are not only totally inadequate but limited to regional availability and cost. Thus, it's clear that a combination of genetic, developmental, psychological, and environmental, socio‐economic factors contributes to the onset and suboptimal treatment of depressive disorders.

## 3 CURRENT DISEASE STATE AND DIAGNOSTIC CRITERIA

Depression is a very common medical condition that is associated with a wide range of emotional, cognitive, and physical symptoms. Depressive disorders involve all major bodily functions, mood, and thoughts, affecting the ways in which a depressed individual, eats and feel about themselves, and thinks. Without treatment, depressive symptoms can last for weeks, months, or a life‐time. Measured by years that people spend disabled with depression, it is the biggest blight on human society—bar none. Research has struggled to lift the “Black Dog,” with more than 350 million people still suffer from the illness every day.

Antidepressant treatment can help some 30%‐40% individuals suffering from depression, with increasing number of treatment options have become available over the past 30 years for individuals with major depressive disorder (MDD). Accompanied by a growing body of evidence‐based medicine describing their effectiveness, efficacy, and safety has provided clinicians with options to determine the most appropriate treatment for each patient as recently highlighted in the Cipriani paper in the Lancet 2018 and other recent publications,^3^ but still leaves the vast majority of patients inadequately treated.

Depressive disorders exhibit different phenotypes and comorbidities, with variations in the number of symptoms, their severity, and persistence according to the previous DSM‐III, DSM‐IV and ICD‐10 classifications and more recently with the revised DSM‐5 and ICD‐10 and 11 classifications (Table 1).^21^, ^22^ Although these classifications have varying degrees of overlap and distinguishing features, their goal is to try and accurately classify the burden of patients suffering from mental disorders. However, the ferocious rhetoric regarding previous and the more recent DSM‐5 and International Classification of Disease (ICD‐11) classification—promises and pitfalls is well documented with regard to the many flaws and discrepancies (see DSM‐5 Pros and Cons.).^23^, ^24^ In spite of the fact of the many changes and improvements in DSM‐5 and ICD‐11 from their predecessors, they both remain subjective categorical classification systems that are fundamentally descriptive in nature, based primarily on self‐reported symptoms, clinically signs with observer bias and few supportive tests (eg, of intellectual functioning). The fact that since the early 1980's, research bodies e.g. NIMH and other funding agencies had virtually mandated the use of DSM or ICD diagnostic categories was argued as a major part of the problem. The DSM “Bible” was seen as dictating US mental health questioning its validity and widely denounced. What was needed was innovative thinking away from symptomatology‐based diagnosis to an alternative approach. In 2009 the NIMH initiated the Research Domain Criteria (RDoc) project was deemed necessary, given the nascent state of the science of mental disorders and the conceptual and empirical constraints of research based on current classifications. The call was that research needed to break out from the straitjacket of current diagnosis.

**Table 1:** Abridged Classification of depressive states

Classification used in guideline	DSM‐IV (code)	ICD‐10 (code)	DSM‐5^a^
Major depression	Major depressive episode, single episode, or recurrent (296)	Depressive episode – severe (F32.2), moderate (F32.1), or mild with at least 5 symptoms (F32.0)	Bereavement exclusion
		Recurrent depressive disorder current episode severe (F33.2), moderate (F33.1) or mild with at least 5 symptoms (F33.0)	Chronic depressive disorders dropped Now classed as Persistent Depressive Disorders
Milder depression	Depressive disorder not otherwise specified (311)	Depressive episode—mild with symptoms (F32.0)	
		Recurrent depressive disorder current episode mild with symptoms (F33.0)	Chronic depressive disorders dropped
		Mixed anxiety and depressive disorder (F41.2)	Anxious distress is now a specifier for unipolar and bipolar and separated into 4 chapters; phobias, OCD, Trauma related, Dissociative disorders
	Adjustment disorder with depressed mood/mixed anxiety and depressed mood (309)	Adjustment disorder—depressive reaction/mixed and depressive reaction (F43.2)	Disruptive Mood Dysregulation Disorder (DMDD)
		Other mood (affective) disorders (F38)	
Dysthymia	Dysthymia (300.4)	Dysthymia (F34.21)	Changed to Persistent Depressive Disorder

a DSM‐5 has several new diagnoses that were not envisioned when ICD‐10/11 were being created and are now mapped into ICD‐9,10. The transition from ICD‐10 to ICD‐11 codes represents an increase from 14 400 codes to 50 000 and not surprisingly with some discrepancies (Abridged version, see links below for various detailed revision of the American Psychiatric Association's Diagnostic and Statistics Manual. https://dsm.psychiatryonline.org/doi/book/10.1176/appi.books.9780890425596 and see 11th Revision of the International Classification of Disease, https://www.who.int/mental_health/management/depression/en/. DSM‐11 has not been included in the table as it is yet to be adopted by WHO and still under review and integrated into DSM‐5. For a list of revised symptoms, see the abridged DSM‐5 criteria below and DSM‐5 Update (August 2015), pages 1‐26. Published by American Psychiatric Association 2016.^21^, ^22^

The development of basic translational science applied to depression and other mental disorders responded slowly to the difficulties of the categorical classification system and represented a long‐term NIMH endeavour. What the NIMH RDoc initiative brought to the forefront was the idea that to understand mental illness in all its complexity, the neuroscience field needs a research framework that accommodates the study of all causal factors together. This was acknowledged to be a long‐haul and there are no right answers that this framework will work. The notion that neural‐circuit based framework will ultimately deepen our understanding of the neurological, biological, psychological, social and cultural structures, and processes that underlie depression and mental illness will ultimately lead to a move away from an out‐dated, systematic biases clinical trial methodology.^8^, ^25^ Accordingly, Thomas Insel in proposing the NIMH's reorientation away from DSM categories stated, “We cannot succeed if we use DSM categories as the gold standard.”

## 4 MAJOR DEPRESSIVE DISORDER: DSM‐5 SYMPTOMS OR ENDOPHENOTYPES?

The symptom criteria for major depression according to the recent DSM‐5 and ICD‐11 guidelines are reported to be very similar although the coding systems are different.

The DSM‐5 (Diagnostic and Statistical Manual of Mental Disorders, 5^th^ Edition) has focussed on more attention gender‐specific factors across disorders, cultural and cross‐cultural assessments, Thus, the multi‐axial system of psychiatric classification (ie, DSM‐III, DSM‐IV TF and ICD‐10 see Table 1) is to be gradually replaced for all psychiatric and mental disorders that are now to be considered on a single axis. For example, in mood disorders the separation of bipolar and related disorders (BPAD) is a major change in diagnostic criteria and clinical descriptions forming a separate chapter for bipolar (affective) disorders (BPAD) in DSM‐5 (see comprehensive reviews on BPAD in references^8^, ^26^, ^27^).

In the case of depression, there are now 8 specific depressive disorders (single‐axis) described in the DSM‐5 (see below). With the aim of increasing the focus on these individual (“personalized”) disorders, their severity, phenotypes/genotypes, and application of numerous specifiers to capture significant advances in clinical research, including advances in neurobiology and genetics.^28^, ^29^

1. Major Depressive Disorder (MDD)
2. Disruptive mood dysregulation disorder (DMDD)
3. Persistent depressive disorder (previously called Dysthymia)
4. Premenstrual dysphoric disorder (PMDD)
5. Substance/medication‐induced depressive disorder
6. Depressive disorder due to another medical condition
7. Other specified depressive disorder
8. Unspecified depressive disorder

Adapted from: American Psychiatric Association (2013).^21^, ^28^

Whereas, DSM‐IV comprised of additional subcategories for catatonic, melancholic, and atypical features and for postpartum onset. Both DSM‐IV and ICD‐10 present affective disorders together in one section, distinguishing bipolar (BPAD) from unipolar disorder (MDD), including dysthymia (see Table 1). Operational problems often encounted with ICD‐10 include complexity, use of different clinical and research definitions, emphasis on single versus recurrent episodes, and the lack of some clinically useful subtypes. Whereas, DSM‐IV assigns separate unjustified categories of medical and substance‐induced mood disorders and failed to code its useful qualifiers,^30^ which now come under separate categories in DSM‐5.

Also, within DSM‐IV, bipolar disorder described a spectrum of disorders in which episodes of depression and mania occur, interspersed with periods of normal mood. Bipolar depression or manic depression (Table 1). BPAD is characterized by cycles of mania and depression, which cause a person with bipolar disorder to experience severe mood swings This has all now changed with the introduction of DSM‐5 (Tables 1 and 2).

**Table 2:** Abridged DSM‐IV criteria for major depressive episode

A Over the last 2 weeks, of the following features should be present most of the day, or nearly every day (must include 1 or 2): a Depressed mood b Loss of interest or pleasure in almost all activities c Significant weight loss or gain (more than 5% change in 1 month) or an increase or decrease in appetite nearly every day d Insomnia or hypersomnia e Psychomotor agitation or retardation (observable by others) f Fatigue or loss of energy g Feelings of worthlessness or excessive or inappropriate guilt (not merely self‐reproach about being sick) h Diminished ability to think or concentrate, or indecisiveness (either by subjective account or observation of others) i Recurrent thoughts of death (not just fear of dying), or suicidal ideation, or a suicide attempt, or a specific plan for committing suicide B The symptoms cause clinically significant distress or impairment in functioning. C The symptoms are not due to a physical/organic factor or illness. The symptoms are not better explained by bereavement (although this can be complicated by major depression)

Although, antidepressants are sometimes prescribed for the treatment of BPAD, lithium, anticonvulsants, valproate, benzodiazepine, atypical antipsychotics (eg, clozapine, olanzapine, ziprasidone, and aripiprazole) are the preferred treatment of choice.

Thus, given the low expectation of clinical success for novel antidepressant drugs over the last 3 decades the notion that the multi‐axial classification of DSM‐IV/ICD‐10 and its predecessors added to the complexity of the diagnostic classification of MDD, its comorbidity with other psychiatric, neurological disorders, and other clinical disorders (eg, cardiovascular disease) clearly contributed to a high chance of failure in heterogeneous depressed patient populations.

After decades of controversy in their development the advent of the revised single axis DSM‐5/ICD‐11 diagnostic criteria for depressive disorders was envisaged would improve clinical success of innovative therapies. However, it appears that this is not the case and may exacerbate the problems with clinical diagnosis. The new classification(s) are under fierce controversy and a torrent of criticism and detractors a (see DSM‐5 Pros and Cons.).^23^, ^24^ The notion is that a number of the DSM‐5 veterans may have contributed to ICD‐11, following the DSM‐5 template and repeating previous mistakes? To add to the confusion and controversy many of the DSM‐5 criteria are still mapped to their outdated DSM predecessors and this may remain so for the foreseeable future?

The introduction of the ICD‐11 diagnostic criteria for depressive disorders is schedule for adoption by the WHO Assembly in May 2019 and by member states in 2022. Given that ICD‐10 was not implemented in the US until 2015, 21 years after its release in 1994?

Therefore, the urgent and timely need to align workable (international) diagnostic criteria along with the rapid advances in neurobiology; pharmacogenomics, disease targeted biomarkers and thereby moving current classifications from subjective behavioral criteria to a more neurobiological emphasis would appear to offer little comfort for the patient in the short‐term (see below). As it stands, the future success of antidepressant treatment for depressive disorders will be largely based on a combination of the controversial DSM‐5/ICD‐11 and differentiation of depressive disorders into defined endophenotypes will be largely based on epistatic data‐driven neurobiology. Some would argue that little has changed and if this is the neurobiological view of the future, why have so many neurobiological hypotheses failed so dismally in the past? The reorientation of our thinking may lie in current revolution in neurobiological techniques and a paradigm shift in our understanding of the neuropathophysiology of aberrant neural circuitry, particularly in the case of MDD in identifying changes in hippocampal brain structures (see later section) due to an impairment of neurogenesis/neuroinflammation.^31^ Using noninvasive technologies, we are now redefining the underlying pathophysiology of depression and final common pathway(s), whereby antidepressants exert their action.^32^ Thus, the hypothesis failures of the past may therefore represent a protracted learning curve resulting from past failures and a naive understanding of complex brain neurochemistry and multi‐modal brain neurocircuitry. And, applying this to ill‐defined diagnostic criteria within heterogeneous patient populations to unmask the so‐called “final common pathway” for depressive disorders.

The hypothesis failures in neurobiological and clinical studies of the past will be reviewed in the next section.

## 5 HYPOTHESIS FAILURE: WHAT WE HAVE LEARNT FROM THE PAST, IF ANYTHING?

**Figure 1:**
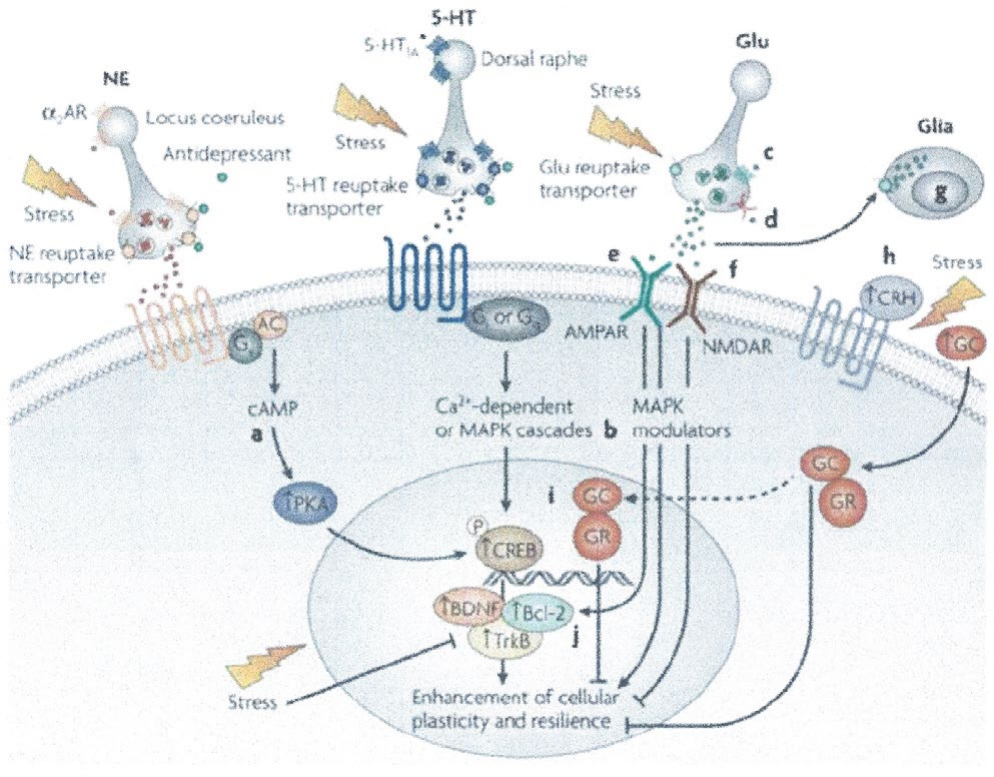
Cellular targets for the development of novel agents for the treatment of mood disorders. This figure shows the multiple targets by which transcription, neuroplasticity, and cellular resilience can be increased in mood disorders. (a) Phosphodiesterase inhibitors increase levels of pCREB; (b) MAP kinase modulators increase the expression of the of the major neurotrophic protein Bcl‐2; (c) mGluR II/III agonists modulate release of excessive levels of glutamate; (d) drugs such as lamotrigine and riluzole act on Na+ channels to attenuate glutamate release; (g) AMPA potentiators up regulate the expression of BDNF; (f) NMDA antagonists like ketamine, esketamine, and memantine enhance plasticity and cell survival; (g) novel drugs to enhance glial release of trophic factors and clear excessive glutamate may have utility for the treatment of depressive disorders; (h) CRF antagonists and (i) glucocorticoid antagonists attenuate the deleterious effects of hypercortisolemia, and CRF antagonists may exert other beneficial effects in the treatment of depression via non‐HPA mechanisms; (j) agents which upregulate Bcl‐2 (eg, pramipexole, shown to be effective in dipolar depression). These distinct pathways have convergent effects on the cellular processes such as bioenergetics (energy metabolism), neuroplasticity, neurogenesis, cellular resilience, and survival. Modified and reproduced from Blackburn^9^

The etiology of depression is unknown. Depression is polygenic in nature with both genetic and epigenetic components, making the use of genetically engineered animal as models for drug discovery unrealistic.^8^, ^9^ This along with our emerging understanding of the complex biochemical mechanisms is compromised by the fact that most of the drugs used to treat depression and other neuropsychiatric disorders (eg, lithium and antidepressants in general) have ill‐defined pleiotropic mechanisms of action with new signaling pathways and neuronal networks being identified, pointing to no “final common pathway” in the mode of action of antidepressant agents Figure 1.^8^, ^32^, ^33^

## 6 THE MONOAMINE THEORY OF DEPRESSION**—**OF LIMITED SUCCESS OR A DISMAL FAILURE?

The longest‐standing theory of depression is based on monoamine dysfunction and drugs acting on monoamine neurotransmission which has dominated the treatment of depression for over 50 years, albeit much maligned in recent times as a too simplistic and may have misguided our understanding of the complexity of the disorder.^8^, ^32^ The fact remains, however, that the monoamine reuptake inhibitors and the MAOI's were shown to have antidepressant activity albeit by chance clinical observations and the discoveries of their modes of action were instrumental in developing the monoamine theory.^8^ In the days when the monoamine theory of depression was evolving, the focus was more on norepinephrine (NE) than 5‐HT (5‐hydroxytryptamine) or dopamine (DA). The theory developed from observations that reserpine depleted monoamines and caused depression, whereas the MAOI's and monoamine reuptake inhibitors enhanced monoamine function and thereby relieved depression. This hypothesis, as well as others discussed below, where the cornerstone of pharmaceutical research for decades.

Over 4 decades the therapeutic goal was to find, a fast‐acting antidepressant. However, this was contended by Duman and a number of groups, that this approach may not be possible based on their neurogenesis hypothesis of antidepressant efficacy.^32^, ^34^ To discover an antidepressant that has an effect within days rather than weeks has challenged researchers for decades to understand the reasons for the delay in onset of the antidepressant action. One theory based on the action of SSRI's is that inhibition of 5HT reuptake initially causes activation of the presynaptic 5HT_1A_ receptors on the cell bodies in the dorsal and median raphé nucleus.^8^, ^9^ This inhibits the firing of 5‐HT neurons, so reducing rather than increasing the release of 5HT from the terminals.^8^, ^9^ According to this hypothesis first proposed as the primary mechanism of action of SSRI's due to an increased activation of 5‐HT postsynaptic receptors in the forebrain and is not achieved until the raphé somatodendritic 5‐HT_1A_ receptors become downregulated or desensitized. However, clinical molecular imaging and postmortem studies failed to find consistent evidence supporting alterations of in patients with MDD.^35^ Furthermore, 5‐HT_1A_ receptor antagonists also failed to achieve consistent clinical efficacy.^8^

Several lines of evidence exist that suggested increased synaptic 5‐HT, and or NE, DA does not account fully for the antidepressant efficacy; (a) rapid increase in synaptic levels of 5‐HT concentration is inconsistent with a rapid response, (b) lowering the concentration of 5‐HT in the synaptic cleft with 5‐HT depleting agents or enhancers failed to induce depression in healthy subjects,^8^, ^9^ (c) long‐term antidepressant treatment cause a reduction in total 5‐HT content in the brain, (d) genetic variants of 5‐HT alleles associated with the potentiation of 5‐HT SERT function (*l allele* 5HTTLPR) has been associated with a reduced risk of depression than variants associated with a decreased SERT function. (*s allele* 5HTTLPR).^32^

Thus, the notion that depression is caused by a deficiency of 5‐HT has now been questioned by several leading groups in the field, as there is no clear evidence that the monoamine deficiency totally accounts for depression and questions the efficacy of monoamine‐based agents.^8^, ^32^ The question remains is there a single unifying mechanism underlying the complex manifestations of depression (MDD)?

In the case of MDD, genetic factors account for about 30% of the variance and environmental factors play a major role in inducing the illness.^36^ The first direct evidence of the importance of variation in drug response was shown in depressed patients with a short form of the SERT promoter, who had a worse response to SSRI's than those with the long isoform.^8^, ^32^ Other genes have been associated with antidepressant treatment and undoubtedly the field of pharmacogenomics and its application to the pathophysiological mechanisms of depressive disorders will continue to grow based on vulnerability gene environment interaction and experience‐dependent biological systems that act cumulatively (eg, chronic stress) throughout an individual's lifetime.^29^ That being said, the impact of genetics on mental disorders over the last few decades have been disappointing, despite the enthusiasm for new era of personalized medicine and an individual's genome. However, emerging results as discussed later may offer hope for future drug therapy based on endophenotype.^29^

## 7 THE FAILURE OF EXPERIMENTAL DISEASE MODELS OF DEPRESSIVE DISORDERS?

Drug discovery in depression has been hampered by the lack of an universally accepted phenotypic screens—animal model(s) that can be used to screen NCEs for antidepressant ‐like effects. Animal models of depression have provided insights into mechanisms associated with MDD endophenotypes but how these models apply to human mental illness and its treatments remains difficult to assess. Although there are several animal models that reproduce some features of depression in the context of stress and/or maternal separation, it is questionable as to whether these are relevant to the human disorder MDD or BPAD. The advantages and disadvantages of animal models for depression are summarized in various comprehensive reviews.^8^, ^37^ However, in many cases, the behavioral features can be reversed by conventional antidepressant drug treatment. Despite this holistic notion and their intrinsic limitations, the full potential of these models has not yet been realized and they represent an underexplored opportunity. The heuristic value and the knowledge gain from behavioral animal models in psychopharmacology are, explicitly or implicitly, the central preoccupation of psychopharmacologists.^8^, ^9^ There are a number of compelling reasons to believe in the legitimacy of animal models in the development of new improved drugs for the treatment of mental disorders; however, these models need to be based on the following criteria.^8^, ^9^, ^33^

- Predictive validity: the ability of a model to accurately predict clinical efficacy of a psychoactive pharmacological agent.
- Face validity: the similarity of the model to clinical manifestations of phenomenon/disorder in terms of major behavioral and/or physiological symptoms and etiology.
- Construct validity: the strength of the theoretical rationale upon which the model is based

Animal models have been defined as experimental preparations developed in one species for the purpose of studying or understanding a phenomenon occurring in another species (eg, the “5‐HT Syndrome” crosses a number of mammalian species).

In the case of animal models of human psychopathology, the aim is to develop syndromes that resemble those in humans in order to study selected aspects of neuropsychopharmacology. The behavioral models are explicitly related to a broader body of theory, as they fulfill a valuable function in forcing the clinician and psychopharmacologists alike to critically examine their assumptions of the manifestations and pathophysiology of depression and bipolar disorders. Importantly, they are still required to provide guidance on optimal dose level selection for clinical regulatory safety, general toxicological, and efficacy studies in humans.^33^

To disparage phenotypic animal models of psychiatric disorders seems unwise today when many molecular manipulations (eg, CRISPR/Cas9 and DAT‐Cre gene editing, CART technology, optogenics, RNA interference, antisense, and viral vector delivery technique), are emerging as a potential paradigm shift in identifying novel neurocircuits and drug targets. And, argues for their construct validity in creating genotype/phenotype models of mental disorders. Endophenotypic screening technology is revolutionizing drug discovery, such as CRISPR‐based, multiple gRNAs can make multiple cuts to multiple genes simultaneously. The ability to do this for polygenic disorders could be revolutionary and a game‐changer for the treatment of psychiatric disorders.

It is clear to all that the etiology of psychiatric disorders is still in its infancy; however, a healthy skepticism provides a valuable service in pointing out the many shortcomings when animal models are measured against the complexities of human behavior, more often or not when aligned to highly subjective clinical data.^8^

However, as new targets emerge through hypothesis‐driven research or serendipity, the challenge is to link the mechanism to a clinical complex and heterogeneous disorder. Consequently, much of the animal research today is framed around physiological and neurobiological phenomena that may bear little resemblance to the disease state. It has long been argued, that the poverty of reliable clinical science feedback needs to be addressed first, which would aid future model development.

In animal studies it is very difficult if not impossible to differentiate among different types of receptors. RNA interference (RNAi) allows posttranscriptional gene silencing where double‐stranded RNA induces degradation of the homologous endogenous transcripts, mimicking the effect of the reduction or loss of gene activity. This technique, therefore, holds promise in understanding hippocampal autophagy.^33^ Recent siRNA‐mediated knockdown of the SERT in the adult mouse and rat brain would support the concept, although selectivity and side effects remain an issue.^33^, ^38^

Other recent methodologies include, antidepressant drug “signatures” using pharmacodynamic EEG measurements in animals and human studies as a measure of “antidepressant efficacy” and more recently with pharmacodynamic changes in EEG gamma oscillations.^39^, ^40^

Finally, the rapid progress in mutated mice studies using CRISPR/Cas9 gene editing technology,^41^ has shown that that differentiation of receptor subtypes can now be achieved for example, the delta subtype GABA_A_ receptor contributes to the antidepressant effects observed in these mice. Supporting, the positive antidepressant activity observed in Phase II/III clinical studies in postpartum depression (PPD) with the GABA_A_ positive allosteric agonist (PAM) brexanolone‐ SAGE ‐547.^42^ The FDA recently approved this intravenous drug, as the first treatment for PPD(https://www.fda.gov/NewsEvents/Newsroom/PressAnnouncements/ucm633919.htm). A second SAGE compound is currently in a Phase III, study which is a more bioavailable inhibitory pregnane neurosteroid analogue ‐ SAGE‐217 *(3*α*‐Hydroxy‐3*β*‐methyl‐21‐(4‐cyano‐1H‐pyrazol‐1’‐yl)‐19‐nor‐5*β*‐pregnan‐20‐one; 3*β*‐Methyl‐21‐(4‐cyano‐1H‐pyrazol‐1’‐yl)‐19‐norpregnanolone;3*α*‐Hydroxy‐3*β*‐methyl‐5*β*‐dihydro‐21‐(4‐cyano‐1H‐pyrazol‐1’‐yl)‐19‐norprogesterone)*, also reported to show significant efficacy in a Phase III PPD clinical study, is now showing promise in a PhaseII/III study for MDD. Thus, along with these recent positive clinical findings and other major advances in gene editing technology, CART (Cocaine‐and‐Amphetamine‐Regulated Transcription) peptide technology, sphingolipid‐controlled autophagy,^43^ represent important targets that are rapidly increasing our understanding of neural circuits in stratifying patient populations based on biological phenotypes. And, in extreme pharmacological responsiveness to psychostimulant‐induced depression may well open up new avenues of research into the underlying pathophysiology of specific depressive disorders.^44^

## 8 OTHER NEUROCHEMICAL THEORIES OF DEPRESSION

### 8.1 Neurogenesis: creation of new neurones critical to antidepressant action?

Antidepressant treatments, such as SSRIs and electroconvulsive shock (equivalent to human electroconvulsive therapy, ECT) increase neurogenesis specifically in the hippocampus.^45^ In fact, the maturation period for neurogenesis in the dentate gyrus appears consistent with the delay for the full therapeutic effects of antidepressants, as previous reported in the seminal work of Duman.^46^, ^47^, ^48^ Thus, these preclinical findings suggest that adult neurogenesis may be modulated by factors associated with MDD, including chronic stress,^49^ and activation of the HPA axis.^50^ While the evidence reviewed above suggests the presence of a link between reduced hippocampal adult neurogenesis and MDD, preclinical and clinical studies have also reported findings that are inconsistent with this hypothesis.^51^

While chronic fluoxetine treatment doubled the number of new hippocampal neurons in normal mice, it had no effect in 5‐HT_1A_ knockout mice. The tricyclic imipramine boosted neurogenesis in both types of mice, indicating that the 5HT_1A_ receptor is required for neurogenesis induced by fluoxetine but not imipramine. Chronic treatment with a 5HT_1A_ selective drug confirmed that activating the 5HT_1A_ receptor is sufficient to spur cell proliferation. An extension of this work using the SSRI fluoxetine in a transgenic cell line from dentate gyrus showed that the SSRI does not affect division of stem‐like cells but increases division of amplifying neuroprogenitor cells that results in new neurons in dentate gyrus. This effect was specific for dentate gyrus.^52^ These results suggest that strategies aimed at stimulating hippocampal neurogenesis provide novel avenues for the treatment of anxiety and depressive disorders. However, the “Holy Grail” of current treatment strategies is to develop antidepressants with a fast onset of action. In this light, the neurogenesis hypothesis would therefore not support this approach. However, this theory is now in question with regard to the fast‐onset and long duration of efficacy observed with ketamine‐like agents (see later and Table 3).

**Table 3:** Types bipolar affective disorder (BPAD)^a^ according to DSM‐5

Bipolar I (BPI) (DSM‐IV 296.00‐296.06, 296.40‐296.7) ☐ 1% prevalence ☐ at least 1 episode of full‐blown mania ☐ episodes of hypomania, mixed states and depression DSM‐5 – BPI &BPII Hypomanic Episode, Criterion F (eg, episodes not attributable drug abuse, a medication, or other treatments) Bipolar II (BPII) (DSM‐IV 296.89) ☐ 0.5%‐3% prevalence ☐ at least one episode of depression ☐ at least one episode of hypomania DSM‐5 – With Melancholic Features and with Atypical Features Specifiers (eg, with mood‐congruent psychotic features and rapid cycling) DSM‐5 ‐ With Season Pattern Specifier (eg, seasonally linked psychosocial stress, loss of energy) DSM ‐5 – Severity Specifier (e.g. to separate “mild”, “moderate”, and “severe”) Bipolar spectrum disorder (DSM‐IV includes all of above plus 3021.13) ☐ up to 5% prevalence ☐ includes BPI, BPII, schizoaffective disorders, cyclothymia

a BAPD is now a new diagnostic criterion in DSM‐5. Major changes are increased energy is now a criterion choice (which is absent in the DSM‐IV criteria above). Mixed features capture subthreshold states and no longer requires full criteria of MDD episodes and concurrent manic episodes. It is beyond the scope of this review to comprehensively cover all the changes from DSM‐IV to DSM‐5 and the reader is referred DSM‐5 Update (August 2015), pages 1‐26. Abridged and modified version, See American Psychiatric Association 2016, for a more detailed information.

### 8.2 Corticotropin Releasing Factor (CRF): CRF_1_ Receptor Antagonists

Corticotropin‐releasing factor receptor antagonists have been sought since the stress‐secreted peptide was isolated in 1981.^53^ Pharmacological and transgenic studies show that brain and pituitary CRF_1_ receptors mediate endocrine, behavioral, and autonomic responses to stress.^54^ Thus, the therapeutic utility of CRF1 antagonists soon became clear and several small molecules progressed into clinical development for depression and other stress‐related indications.^55^ However, data with small‐molecule CRF_1_ antagonists did not consistently shown efficacy in animal models of “antidepressant‐like” activity.^55^ And, in spite of numerous studies no subsequent CRF_1_(or _2_) antagonist has successfully completed a definitive Phase III clinical trial, including at least 3 drugs for MDD; Verucesfont ‐GSK561679, GW876008, and Pexacerfont – BMS562,086.^55^, ^56^

## 9 GLUTAMATE, KETAMINE, AND N‐METHYL‐D‐ ASPARTATE (NMDA) RECEPTOR ANTAGONISTS

Ketamine and NMDA receptor antagonists have recently demonstrated potential in the treatment of depression with rapid and positive results in patients with suicidal ideation or facing treatment‐resistant depression and are considered to be “next generation” antidepressants.^57^, ^58^

**Figure 2:**
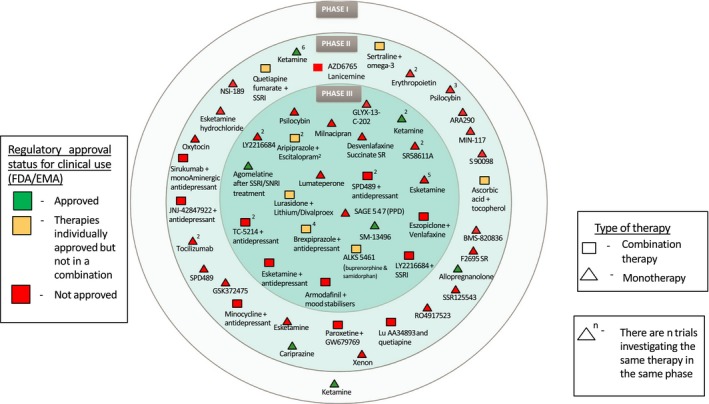
Agents in NCT clinical trials for the treatment of depression 2018 (from FDA clinicaltrials.gov (https://clinicaltrials.gov) register and EUDRA CT European trails register(https://www.clinicaltrialsregister.eu.).September, 2018). Phase III drugs—Esketamine (Spravato) for treatment resistant depression and SAGE 547 (brexanolone injection, Zulresso) for postpartum depression (PPD) both approved by FDA in 1Q2019. My thanks to Dominic Cage for help with the analysis

While it rapidly treats depressed patients, ketamine has pronounced psychotomimetic effects (symptoms of psychosis and dissociative behavior), and it can only be administered intravenously. The s‐enantiomer of ketamine, esketamine, however, has greater affinity at the NMDA receptor and is showing promise in depression following intranasal administration (INDD) To fully determine clinical efficacy and overcome patient tolerability and compliance, 2 studies in depressed (MDD) patients with intranasal esketamine have recently reported and gained FDA approval (see Figure 2). A Phase II/III study in patients with treatment resistant depression and no suicidal ideation NCT02418585 (https://www.janssen.com/new-phase-3-data-show-esketamine-nasal-spray-demonstrated-rapid-improvements-depressive-symptoms), and a Phase II study in the treatment resistant depression with no immediate suicidal risk ‐ NCT02133001,

(https://www.janssen.com/newly-published-phase-2-study-found-esketamine-demonstrated-significantly-rapid-improvements)

The positive findings in these studies for treatment resistant depression and rapid‐onset have led to several ongoing studies (Figure 2, Table 4), which may to lead to a breakthrough therapy, although recent studies from Stanford suggest that other mechanisms (opioid) may account for the efficacy of this class of compounds that will challenge the design of future clinical studies with ketamine and ketamine‐like compounds.^59^ To further add to the complexity of this hypothesis, other reports suggest ketamine‐like drugs may have antidepressant properties partly by regulating monoamine signaling,^60^ inflammatory systems,^61^ and possible epigenetic mechanisms.^36^, ^62^

**Table 4:** Ketamine‐like drugs

Target Mechanism	Compound	Clinical Trial Phase	Sponsor
NMDR (nonselective)	AXS‐05	III	Axsome Therapeuitics
NMDAR Antagonist (nonselective)	AVP‐786	II	Avanir/Otsuka
NMDAR Antagonist (nonselective)	Esketamine (intranasal)	III	JNJ/Janssen
NR2B Subunit	CERC‐301	II	Cerecor
NR2B Subunit (Modulator with GlyB site (partial agonist)	GLYX‐13/NRX‐1074 Rapastinel (IV)	III	Allergan
NR2B Subunit	AV‐101	II	VistaGen

NMDAR, N‐methyl‐D‐aspartate receptor, NR2B, N‐methyl D‐aspartate receptor subtype B.

One example of recent drug, in this field is AV‐101 (4‐Chlorkynurenine), reported to be an oral N‐methyl‐D‐aspartate (NMDA) receptor and glycine B (GlyB) antagonist in Phase II clinical development—initially as a new adjunctive treatment of MDD in patients with an inadequate response to current FDA‐approved antidepressants and treatment‐resistant patients (see Figure 1 & Table 3). AV‐101 does not block NMDA receptor activity but inhibits and modulates it through specific GlyB binding site and activates AMPA pathways. Interestingly, this compound was shown to have ketamine‐like properties in animal models.

Another compound sharing many of the same pharmacological effects of ketamine, Lanicemine (AZD6765) showed no significant difference between lanicemine and placebo on any outcome measures related to MDD in large randomized, placebo‐controlled study.^63^

AV‐101 as Adjunct Antidepressant Therapy in Patients with Major Depression (ELEVATE) NCT03078322 Antidepressant Effects of the Glycine Receptor Antagonist AV‐101 (4‐chlorokynurenine) in Major Depressive Disorder NCT02484456.

https://clinicaltrials.gov/ct2/show/NCT03078322?term=AV-101&cond=MDD&rank=1

https://clinicaltrials.gov/ct2/show/NCT02484456?term=AV-101&cond=MDD&rank=2

Thus, with other clinical data still awaited the jury is still out for ketamine‐like drugs being the “next generation” of rapidly acting antidepressant drugs Table 4. (see.,^64^ for an excellent review of glutamate signaling in depression).

In summary, given the overall complexity of the underlying neurochemical changes attribute to the pathophysiology of depressive disorders and the recent single axis DSM‐5 classification of depressive disorders, raises questions concerning the validity “fit‐for‐purpose” animal models of depression and their ability to mimic monoaminergic and non‐monoamine approaches in antidepressant drug development. In retrospect, despite the criticism and controversy attached to them, which is largely based on subjective assessment criteria much like categorical classification systems that are fundamentally descriptive—as described in the various complex DSM/ICD “specifier” criterion. Therefore, some would argue that with insufficient evidence at present to align emerging neurobiological technologies related to animal models, disease pathologies/neurocircuitry and endophenotypes, it will take time before they eventually become congruent and play an increasing part in antidepressant drug development in the future (see above).

The light at the end of the tunnel for animal models will depend on the technological advances in gene editing, optogenetics and a greater understanding of neuronal networks in the hope that the continued confluence of neurochemical hypotheses will lead to a greater understanding of neurocircuitry pathways, translational pharmacology and less failures of compounds currently in clinical development^65^ see Figure 1.

## 10 CLINICAL TRIAL FAILURES AND FUTURE CHALLENGES

It is clear that not all individuals with depression or undergoing episodes of depressive disorders according to current diagnostic criteria experience every depressive symptom, with severity and episodes varying between individuals and over time. Apparently, in the clinical setting it can take up to 2 hrs to fully evaluate a patient under previous subjective DSM IV criteria for MDD and the diagnosis may be different on the second evaluation.

And, where this leaves the Primary Care Physician's (PCPs) in diagnosing depressive disorders in several minutes of consultation is likely to lead to misdiagnoses and prescribing mistakes. Furthermore, impacting on the inclusion and exclusion criteria for many clinical trials for novel agents to treat MDD, compromised by the lack of clarity in diagnosis and potential for misdiagnoses. Thus, adding to the level of complexity and study failure and compounded by the fact that FDA guidelines have only been recently updated (June 2018) since their inception in 1977. Some would argue that little has been done to address such criteria in the selection of patients into studies (see link to FDA Draft Guidance to Industry below for MDD: Developing Drugs for Treatment).^66^ (https://www.fda.gov/downloads/Drugs/GuidanceComplianceRegulatoryInformation/Guidances/UCM611259.pdf).

Therefore, the perceived lack of clarity over 40 years along with inappropriate labeling is changing with new initiatives from the FDA and the European Medicines Agency (EMA) guide lines for investigating antidepressants in MDD. Although, not without their differences, in particular acknowledging that antidepressants may not be effective with mild depression, patients nonetheless are diagnosed with MDD and considered sufficient for registration package for a license for “treatment of Episodes of Major Depression. According to EMA guidelines (see EMA/CHMP/185423/2010 Rev. 2 previously (CPMP/EWP/518/97, Rev. 1). The detection of MDD requires the presence of mood disturbance or loss of interest and pleasure in activities accompanied by at least 2 (ICD‐10) or 4 other symptoms of depression (DSM IV‐TR). These core symptoms may vary from patient to patient; however, they are typically seen for much of the day, almost always every day for at least 2 weeks and are associated with relevant psychological distress and considerable impairment of psychosocial and work functioning. Interestingly, previous DSM‐IV guidelines did not address the logic behind such clear differences and inconsistencies.

With ever increasing numbers of treatment options available for patients with MDD and BPAD, and a growing body of evidence from the pharmaceutical industry describing their potential efficacy and safety, PCPs often find it difficult to determine the best and most appropriate evidence‐based treatment for each patient. Thus, European and US consensus guidelines using statistical methods to synthesize and evaluate data from a number of studies (meta‐analyses) were published for DSM‐IV and ICD‐10 with various recommendations for the treatment of major depression and bipolar disorder.^67^, ^68^, ^69^

The use of meta‐analysis for evidence‐based treatment depends on which study you read as to the efficacy of antidepressant agents, in particular for SSRI's.^2^, ^3^ A number of studies do agree that the magnitude of benefit of antidepressant medication compared to placebo increases with severity of depression symptoms and may be minimal or nonexistent, on average in patients with mild or moderate symptoms. Drug‐placebo difference in antidepressant efficacy increases as a function of baseline severity and can be either relatively small or substantial for severe symptoms, which may account for many clinical efficacy failures.^70^

The previous guideline as discussed, were far from optimal and barely cover the nature and detection of depressive disorders, acute treatment with antidepressant drugs, choice of drug versus alternative treatment, practical issues in prescribing, management when initial treatment fails, maintenance treatment to prevent recurrence, and the increasing importance of discontinuation of treatment.^71^ A recent report indicated that it is not uncommon that withdrawal (discontinuation syndrome) effects from antidepressants can last for several weeks or months,^72^ supporting earlier findings.^73^

These findings are at odds with 2 national guidelines (American Psychiatric Association—APA and National Institute for Health and Care Excellence—NICE, see below for links) being one of a number of criteria for diagnosis of depressive disorders and side effects that appear to be ill‐ defined and clearly out of date. The introduction of the new DSM‐5 & ICD‐11 was hoped to go some way and redefine the treatment of MDD and depressive disorders using single axis criteria. Only time will tell if one blunt instrument has been substituted for another. The key elements of antidepressant drug development are discussed next and how these may impact on clinical success or failure.

https://www.psychiatry.org/psychiatrists/practice/clinical-practice-guidelines

https://www.nice.org.uk/guidance/cg90/chapter/introduction

## 11 CLINICAL EFFICACY

For decades, clinical studies with antidepressants invariably involve self‐reporting of symptoms using standardized questionnaires including the Hamilton Depression Rating (HAMD ‐17) scale for depression (17 or 21 items of a 23‐item scale). With an efficacious drug treatment usually registers at greater than 50% in the baseline HAM‐D score on a 17‐ or 21‐item scale. The Montgomery‐Asberg Depression Rating Scale (MADRAS) is a ‐item scale that is used more often to identify anxiolytic‐like properties. Both rating scales provide a wealth of clinical data to show that all current antidepressants are effective in 20%‐70% of patients treated, with placebo responses occurring in 30%‐50% of treated individuals.^74^

Therefore, the major issue with the current blunted clinical instruments and trial design is that the overall efficacy of antidepressants may be less than 50%. Retrospective analysis of completed antidepressant trials has revealed that 4 out of 6 trials do not differentiate from placebo.^74^ The fact that little is known about the clinical relevance of the HAMD‐17 total scores and how they translate into clinical severity in terms of efficacy and remission from a clinical perspective has only recently been tested by Leucht et al.^75^ The authors questioned “what does the HAMD mean?” They concluded from 43 drug trials in patients with MDD (n = 7131) that a baseline illness of severity was observed when comparing Clinical Global Impression‐severity (CGI‐S) with HAMD‐17 scores.^75^

A more recent study critical of the clinical relevance of the HAMD‐17 diagnosis criteria, argue that frequent using HDRS‐17‐sum scores*,* used as an effect parameter may have distorted the current view on the usefulness of SSRIs and hampered the development of novel antidepressants.^3^ Firstly, these authors argued that HDRS‐17 is multidimensional, indicating that relevant improvement in one domain of symptoms may be masked, due to enhanced variability, by lack of improvement in other less relevant domains. Second, the included symptoms differ in terms of burden of illness and many of them correlate poorly with depression severity. Third, some items refer to several heterogeneous symptoms, and the different grades for a certain item do not always represent differences in severity but qualitatively distinct phenomena; both these aspects may contribute to the poor interrater reliability marring the reliability of the instrument. Fourth, many patients reporting some of the symptoms to be absent already at baseline is bound to reduce the sensitivity of the instrument by enhancing variability, as is the fact that some of the symptoms, such as backaches and headache, are common also in nondepressed subjects, and may therefore be present also after recovery.^3^

Thus, the limitations and challenges of antidepressant clinical trials are well documented and relate to several inherent variables; these include the spontaneous remission observed in the length of the normal 6‐8 week clinical trials and the power of placebo in these studies. The different phenotypes of depression, ranging from mild to severe forms of the illness, add to the “noise” of the trial. To meet regulatory requirements and approval in the US, Europe, and Japan, large clinical trials are required with at least 2500 patients (at a cost of around $15 000/patient based on 2005 figures). Therefore, as long as the “regulators” requirements are for a double‐blind, placebo‐controlled trial with a positive arm, the chances of failure remain far too high. The high cost to risk ratio of such studies have driven many pharmaceutical companies to seek alternative clinical assessment strategies, for example, “fixed versus flexible” dose design and to engage in continuous phenotypic refinement of trial populations to determine patient subsets (stratification) that will improve efficacy scale ratings.^33^ Moreover, the rapid advances in computer technology machine learning and AI use of Bayesian statistics^76^ along with adaptive clinical trial design (see recent FDA draft guide lines—2018 below) will undoubtedly drive the design and outcome measures for more transformative clinical studies in future,^77^ https://www.fda.gov/downloads/drugs/guidances/ucm201790.pdf)

The failure of previous consensus documents that agreed on the use of DSM‐IV‐TR and ICD‐10 criteria in providing guidelines to improve the management and outcome measures of antidepressant trials were clearly in need of urgent revision, as they were suboptimal and decades old. What is more to the point is a recent large‐scale meta‐analysis of antidepressant studies,^3^ it is well recognized that efficacy varies between classes of antidepressants, and the advantages of the newer compounds such as SSRIs is based on their improved side‐effect profile rather than their antidepressant efficacy.^3^ Owing to the widespread use of antidepressants, clinicians now demand that an NCE (New Chemical Entity) has an acceptable safety profile. The incidence of all transient side‐effects, such as nausea, headache, dizziness, agitation, sexual dysfunction, diarrhea, and weight gain, should be measured. Concomitantly, many individuals who are prescribed and use antidepressant medications may not have met criteria for mental disorders. Recent data from the Baltimore Epidemiologic Catchment Area Study indicate that antidepressants are used in the absence of clear evidence‐based indications.^73^, ^78^, ^79^

## 12 CLINICAL TRIAL DESIGN: THE ROLE OF PLACEBO RESPONSE AND OUT COME MEASURES?

Over the last 30 years, the randomized, double‐blind, placebo‐controlled trial (RCT) remains the gold standard for treatment comparisons and still required by some regulatory agencies (see below). However, some European countries do not permit inclusion of a placebo‐controlled group. The role of the placebo response in current and future clinical trial design is in question for the following reasons. The decision of whether to combine data from both placebo‐and nonplacebo‐controlled studies (ie, active‐comparator only trials) is debatable, as the active comparator often fails to show efficacy. Consequently, there is need of urgent reconsideration trial design given the need for “better” antidepressants and diagnosis criteria.^30^, ^69^ It is abundantly clear that the greatest emphasis and cost goes into designing clinical trials, diagnosis, enrolment procedures, and data collection, and we continue to fail miserably on primary and secondary efficacy endpoints.

For example, the primary endpoint for the HAM‐D, the FDA usually requires a minimal meaningful improvement of a 50% decrease to a score of less than 10. Although a rating score reduction in 50% is accepted by the FDA, whereas clinicians consider remission rate as a more meaningful endpoint. Remission rate is defined as a score of less than 12 according to the MADRAS scale at any point in time during the study. The MADRAS score decreases as depression symptoms improve. MADRAS measures the severity of a number of depressive symptoms including mood and sadness, tension, sleep, appetite, energy, concentration, suicidal ideation, and restlessness and is now standard practice in the European Union. The use of secondary endpoints such as the HAM‐A (Anxiety) scale and Clinical Global Impressions Scale (CGS‐1) are included mainly for hypothesis setting or to support the information obtained from primary endpoints.

It is estimated that less than 50% of the active treatment arms showed a significant difference from placebo and the magnitude of the change in the placebo group had a greater influence on the drug‐placebo difference than the change in the drug group. The proportion of trials in which antidepressants were shown to give a significantly better HAM‐D score than placebo (*P* < 0.01) was 59.6% (34/57 trials) for flexible dose trials and 31.4% (11/35) for fixed dose trials. Over the decades researches have questioned such outcome measures and have noted that uni‐dimensional subscales of the HAM‐D are more sensitive to drug‐placebo differences than is the total HAM‐D score.^74^

Treatment effects are often evaluated by comparing change over time. However, valid analyses of longitudinal data can be problematic, particularly if some data are missing for reasons related to outcome measures and drop‐outs. Last Observation Carried Forward (LOCF) protocols are a common method of handling missing data because of their simplicity and conservative nature. Recent advances in statistical theory and their implementation have made methods with far less restrictive assumptions than LOCF readily accessible resulting in the use of likelihood‐based repeated measures approaches, which have a number of theoretical and practical advantages for analysis of longitudinal data and dropouts. A number of methods are gaining acceptance with advances in computer technology since the early 90's (eg, Bayesian statistics) and mixed model repeated measures (MMRM), which has been extensively studied in the context of neuropsychiatry clinical trials.^80^ These studies suggest that MMRM yields 75% empirical power compared with 50% for LOCF. is simple to use, easy to implement, and to specify a priori. It is also more likely than LOCF to give adequate control of type I (false‐positive) and type II (false‐negative) errors. In other words, the use of either MMRM or LOCF will lead to the same conclusions but MMRM is likely to yield fewer mis‐steps along the way according to some groups.^80^ An extension of the MMRM, the novel nonlinear NLMMRM provides a tool for assessing a weighting factor collected from various centres thereby controlling the confounding effect of high placebo response across sites, to increase signal detection and to provide a more reliable estimate of the “true treatment effect” (TE) by controlling false negative results associated with excessively high placebo.^81^

To date, few if any, published comparative study of newer antidepressants has enrolled a sufficiently large group of patients to have the power to reliably detect the differences between 2 effective treatments according to a recent critique.^74^ One exception to this is the NIMH‐sponsored Sequenced Treatment Alternatives to Relieve Depression (STAR*D) project, which enrolled 5000 patients is a comparative treatment trial.^82^, ^83^ Although, some might argue that in the STAR*D study, patients with comorbid disorders and lower levels of depression severity were included with placebo control group, a perceived limitation of the study. And, therefore, no firm conclusion can be drawn on the effectiveness of the medications in this broad population of patients.^83^

Unfortunately, owing to the cost and resources required to conduct studies of sufficient size, the average RCT evaluating antidepressant effects is woefully underpowered. For example, in a recent review of 186 RCTs examining the efficacy and tolerability of amitriptyline in comparison with other antidepressants, the average number of patients per treatment group was 40‐35. In an analysis of pivotal studies (ie, well‐designed, well‐controlled studies on which the FDA bases decisions about the efficacy of NCEs) for 7 newer antidepressants, only 65‐75 patients were included per study arm.^74^ Thus, the average study comparing 2 effective antidepressants would have less than 20% power to find a real, albeit modest (ie, 10%), difference in response rates. Put another way, the likelihood of a false‐negative finding (ie, a type II error) would be 4 times greater than the chance of observing a statistically significant difference.

It is apparent that specific treatment effects have declined in recent decades. This may be due to selection bias at work that differs from that of a generation ago. The sample size, the number of centers, treatment arms, dosing (eg, flexible dosing versus fixed), and different expectation biases all potentially influence results. For example, in the 1960s, more trials evaluated hospitalized patients who are less responsive to placebo and who have a more robust response to antidepressants.^74^ Beyond the issue of inpatient/outpatient status, older studies were more likely to enroll patients with more severe forms of depression, BPAD, psychosis, and recurrent melancholic subtypes of depression. In addition, the efficacy of antidepressant interventions was less well understood then (which may have lowered expectations of the patient or clinician) and fewer potential participants had ever received an effective course of pharmacotherapy.

Contemporary trials, on the other hand, may be enrolling a different population: highly selected ambulatory less severe depressed patients who are often contacted through the mass media. Thus, these subjects may be less severely depressed and are rarely treatment naïve.^74^ Attempts to lessen these problems by restricting enrolment to patients with relatively high levels of pretreatment severity have often, in fact, accentuated them by inadvertently causing an inflation of entry depression scores.^74^ Many clinical trials use entry criteria based in part on a minimum score for the same instrument used to evaluate efficacy. Investigators may be motivated, consciously or not, to increase baseline scores slightly in order to enter subjects into the trial. Such scores may then decrease by that same amount once the subject is entered, thus contributing to what appears to be a placebo effect—if not analyzed appropriately.^74^

Another factor influencing the apparent effectiveness of antidepressants aside from the placebo‐effect is the so‐called “file‐drawer effect”: the bias introduced by the tendency to publish positive but not negative studies. This bias is most evident when comparing reviews of published studies with reports that are based on data sets that have been submitted to the FDA for regulatory review.^74^ For example, on the basis of studies conducted for the registration of new antidepressants from fluoxetine to citalopram the effects of antidepressants appear to be only about half the size (relative to placebo) once the unpublished studies are considered.

## 13 NEW INITIATIVES OF CONDUCTING AND EVALUATING CLINICAL TRIALS

A number of recent encouraging statements from FDA on advancing the development of novel treatments for neurological conditions is part of broader effort on modernizing FDA's new drug review programs. Previously, a number of initiatives designed to evaluate psychiatric medicines included the New Clinical Drug Evaluation Unit Program (NCDEU, now ASCP) funded by the NIMH, engaged over 1000 clinicians and industry and regulatory personnel. The ASCP addressed the question of whether clinical trials of antidepressants reflect drug potential, and several groups involved in the initiative focused on different aspects of trial design, for example, heightened placebo effect from such factors as a high drop‐out rate (survival analysis), poor site selection or poor protocol design, and their effect on masking the potential of active drug.

The importance of controlling the confounding variables in the development of a new antidepressant compound is clear to reduce the failure rate in clinical evaluation. For example, spontaneous improvements of depressive symptoms contribute significantly to the placebo effect. Retrospective analysis showed that in placebo‐controlled depression trials, the placebo effect is more prominent during the single‐blind placebo run‐in phase. The difference was unlikely to be due to different rates of spontaneously improved depression between the 2 trial phases. In addition to validating an NCE, comparisons were made between fixed‐dose clinical studies to establish a minimal effective dose of the new agent and discourage subsequent use of excessively high doses with associated heightened side effects. It is argued that variable dose studies (flexible) are more cost effective when attempting to demonstrate efficacy and reduce treatment failure, whereas fixed‐dose studies require larger sample size and subject the patients to either too low or too high a dose of a novel drug.^84^

## 14 ADAPTIVE CLINICAL DESIGN

Recent innovations in the cancer field has led to real‐time adaptation of on‐going clinical trials based on emerging data, thus provoking a revision of the hypotheses being tested and potentially reducing treatment failures. Treatment arms can be altered, or biomarker strategies adopted, depending on drug efficacy data.^85^ Such studies are a paradigm shift in the cancer field and the evolution of exciting new biomarker data in depressive disorders and other psychiatric indications may soon aid rapid diagnosis by providing the molecular and neurobiology insights to guide accurate prediction of treatment response and efficacy—the advent of precision medicine.^77^, ^86^, ^87^

## 15 RATING SCALES

One of the fundamental issues related to gauging the effectiveness of antidepressants is the choice of a validated rating scale, which is often arbitrary, and that depression symptom scales vary in their psychometric properties, conceptual focus, response burden, and discriminating power. In a study reported as part of the NCDEU sponsored program at Duke University, a community control comparison of 4 scales in 688 patients was conducted (559 patients with major depression and 129 normal volunteers). The Duke study employed different assessment scales including the MADRAS and the Centre for Epidemiologic Studies Depression Scale (CESID), as well as the Carroll Depression Scale (CDS, 52‐item scale) and Brief Carroll Depression Scale (BCDS, 12‐item scale). The findings from this study using meta‐analysis found that the 4 scales intercorrelated highly significantly, diagnostic specificity was high, and mean depression scores for patients were significantly greater than those of the normal subjects. The NCDEU studies concluded that while validated depression scales effectively separated clinically depressed patients from the community control subjects, they vary in the cognitive burden placed on the patient and the effectiveness measure appears less robust when used in primary care, which some would argue that clinical success in well controlled clinical trials does not readily translate readily into clinical practise.^69^

As discussed, assessing the speed of onset of a new antidepressant is critical to the patient compliance and a key differentiation factor in the success of a new antidepressant drug both from the pharmacological standpoint and in terms of techniques and clinical instruments needed to record the speed of onset of novel antidepressant agents. Advances personalized computerized enabling technology (wearable) is rapidly changing the multifactorial measurements and data analysis in many clinical studies (orthogonal data sets). Since the introduction of interactive voice response (IVR) technology over a phone line in the 90's which enabled remote evaluation of treatment response at set times 24 hours day from any touch phone. A new era of clinical instruments technology and machine learning are currently under investigation by regulatory agencies to improve the design, outcome measures, and continuous data analysis in clinical trials for depressive disorders and in other therapeutic areas (personalized medicine) to reduce data errors and trial failures.^85^, ^87^, ^88^

### 15.1 Bridging studies

In a “bridging study,” dosage is optimized early in development by determining the maximum tolerated dose of a compound in patients. Consecutive panels of patients each receive higher doses of study drug until a minimum in‐tolerated dose is reached. The dose immediately below this one is then considered the maximum tolerated dose. Careful subject selection, adequate facilities, and highly qualified, experienced personnel are critical to the successful implementation of a bridging study. Correctly done, bridging can streamline the overall drug development process, while making the Phase II and III trials safer for patients. The International Conference on Harmonization (ICH) ES (1998) guidelines were developed to provide a general framework for evaluating the potential impact of ethnic factors on the acceptability of foreign clinical data to facilitate global drug development and registration of NCEs, and to reduce the number of clinical trials for international approval.

An essential prerequisite for the acceptance of foreign data is a ‘Complete Clinical Data Package’ including foreign data that have to meet all regional regulatory requirements. Furthermore, sponsors were requested to show whether the “foreign clinical data” could be appropriately extrapolated to the new geographical region. The assessment of medicines sensitivity to ethnic factors has to be done according to given intrinsic as well as extrinsic ethnic factors. Supplemental “bridging studies” may become necessary to provide clinical or PD as well as PK data allowing an extrapolation to the population of the new region. Extrapolation via a bridging study can avoid the need to conduct additional, expensive clinical trials in the new region, and can facilitate access to superior treatment to patients in a timely fashion. The technical challenge of a bridging study is to demonstrate “similarity” of profile (extrinsic ethnic factors, such as different medical practice in the new region, were the source of concern in terms of affecting the efficacy and safety of new medicines, statistics, sample size, etc.) of an NCE and increasing the success and diminishing the failure of a new antidepressant drug.

### 15.2 Study trial length

Although there is much controversy in the clinical community, a review of the 6 most commonly prescribed antidepressants found that efficacy studies were virtually always short‐term, rarely exceeding 12 weeks of treatment.^67^, ^68^, ^69^, ^70^, ^71^, ^72^, ^73^, ^74^, ^75^, ^76^, ^77^, ^78^, ^79^ Hence, it can be argued that in the real world, the clinical value derived from such studies remains restricted and suboptimal. Thus, only 18% of the observed changes during these short‐term studies in patients with nonsevere forms of depression could be attributed to the active effects of medication. Active medication and placebo both shared 82% of the maximum clinical changes observed, leading to the conclusion that, assuming the effects of the antidepressant medication and placebo are additives, the effects of the medication, even in this clinically favorable group (very few or no psychotic or suicidal participants, very little comorbidity), is extremely modest, negligible, and potentially of little clinical significance. In summary, such studies strike at the core of our understanding of neuropsychopharmacology drug development. Indeed, there has been considerable debate as to the nature of these analyses and how they can be best interpreted to achieve success rather than failure.^80^, ^81^ However, the single most comforting suggestion for psychopharmacology is that the powerful antidepressant effects of these drugs are actually masked by the inadequacy of current clinical trial designs and that the research strategy for the evaluation of novel psychotropic agents, according to Matthews and colleagues over a decade ago, needs significant rethinking and reevaluation.^8^ One immediate and possible solution to de‐risking clinical trial design failure would be the availability of robust biomarkers but presently there are no approved biomarkers for MDD (see below).^89^

### 15.3 Biomarkers for Depressive Disorders?

The main uses of biomarkers for drug development are:

- discovery and selection of lead NCEs;
- generation of pharmacokinetic (PK) and pharmacodynamic (PD) models;
- aid in clinical trial design and expedite drug development;
- serving as surrogates for clinical or mortality endpoints;
- optimizing drug therapy based on genotypic or phenotypic factors; and
- definition of patient enrolment in studies and help with stratification (biosignature development).

A major factor in the development of “better” antidepressants has been the lack of robust biomarkers, which has seriously limited progress in the treatment of depressive disorders in mitigating the risk of treatment failure. Biomarkers are actively being investigated in psychiatry and neurology through a wide variety of procedures and initiatives. The knowledge gained in the use of biomarkers is currently being integrated into databases for use by the scientific community, for example, Drug companies in partnership with FNIH along with FDA guidelines;

https://fnih.org/what-we-do/biomarkers-consortium/programs/inflammatory-markers-early-detection)

https://www.fda.gov/Drugs/DevelopmentApprovalProcess/DrugDevelopmentToolsQualificationProgram/Biomarker

FNIH Web Announcement (April 26, 2018): The FNIH Biomarkers Consortium Launches Project to Improve Diagnosis and Treatment of Neurodegenerative and Psychiatric Diseases.

The advantages, disadvantages, and limitations of selected biotechnologies for assessing the access of a New Compound Entities (NCE) to the brain are reviewed in.^8^ Brain imaging technology is used to study the interaction of an NCE with its target, making this a preferred technique but only used in specialist centers. Moreover, there is a limited number of targets for which validated positron emission tomography (PET) or single photon emission computed tomography (SPECT) ligands are available. And, the data gained, as reported in a PET study with a neurokinin 1 (NK_1_) receptor antagonist (aprepitant) showed that the NCE had greater than 90% occupancy/target engagement of central NK_1_ receptors and shown to be active in an early Phase II efficacy study of depression but not in a subsequent larger Phase III studies, making this a striking example of a novel target with an excellent PET ligand that fails to be supported by clinical data and thereby questions the entire clinical hypothesis, the PET ligand, and/or inadequate patient stratification/diagnostic/clinical criteria in the Phase III study.^90^ These authors expressed caution should be exercised in the appropriate use of PET occupancy data to select doses for drug development programs in neuropsychiatry. The relationship between exposure, receptor occupancy and clinical response should be established. And, stating that “a crisis of confidence has followed the failure of this and other programs in neuropsychiatry, with a far reaching and detrimental impact on pharmaceutical research.” However, major advances in imaging technology, functional magnetic resonance (fMRI) have recently reported that patients with depression can be divided into 4 neurophysiological subtypes (“biotypes”) defined by distinct patterns of dysfunctional connectivity in limbic frontostriatal networks. Therefore, clustering (stratifying) patients and enabling the development of diagnostic classifiers (biomarkers) that most likely will target specific patient phenotypes.^89^, ^91^ The validation of these assessments against relevant biomarkers, across large multi‐site studies will add to their cogency.

According to Bieck and Potter,^92^ a single approach may not provide the answer to addressing the question of brain penetration and drug efficacy. Instead, a multifactorial approach to biomarkers in CNS disorders may well be the answer, using a combination of imaging technology fMRI and PET (where PET ligands are feasible) and CSF studies. Advances in blood‐based protein biomarkers/micro RNA's and endophenotype‐based approaches are currently in progress for a number of neuropsychiatric disorders.

Progress, however, has been slow in providing disease biomarkers or approved diagnostic tests by regulatory authorities. This is true for major depressive disorder (MDD), despite its prevalence in the general population and the widespread acceptance of its biological basis. Studies using strategies like genome‐wide association (GWAS) and candidate gene analyses have identified a number of biomarkers of MDD, including serum levels of neurotrophic factors, inflammatory cytokines and HPA axis hormones, but none have proven sufficiently powerful or robust enough for clinical use yet.^16^, ^93^ The lack of biologically based tests available for use in identifying subgroups of patients with MDD is a significant impediment to personalized and more effective treatment, because it means diagnosis continues to be driven by subjective symptoms. While genetic studies^16^ of MDD and other depressive disorders have not yet led to diagnostic and treatment biomarkers, progress in determining the role of the genome in drug metabolism heralds the first effort in personalized prescribing for the antidepressants. The FDA suggested and approved genotyping tests for common variants of drug metabolism genes, such as the cytochrome P450s. This is particularly important when prescribing antidepressants with potential drug‐drug interactions. In the future, by using on‐site diagnostic test a physician can select an appropriate antidepressant for a given patient genotype, as differences in clearance, half‐life, and peak blood concentrations are controlled by genetic variability in drug metabolism and are patient specific.

Thus, personalization and microsegment of populations based on the characteristics of the individuals endophenotype in drug choice can be achieved because these tests: (a) identify responders and nonresponders; (b) provide alerts to possible adverse drug events; and (c) help optimize dose. Improved ways of diagnosing and prescribing effective treatments for depressive disorders are urgently needed, as the available methods are inadequate, and symptom based. In the foreseeable future, further interrogation of the genome may serve as the basis for development of new personalized medicine strategies for diagnosis and treatment of MDD.^94^

Although, blood tests remain elusive, advances are being made, for example, elevated morning cortisol is a stratified population‐level biomarker for MDD in boys with high depressive symptoms along with 26 candidate biomarkers and compared their expression in human subjects with and with‐out early onset MDD.^95^ Circulating miRNAs are also being considered as possible endophenotype‐based biomarkers in disease pathogenesis, stratification and in monitoring therapeutic responses because of the presence and/or release of miRNAs in blood cells as well as in other peripheral tissues.^96^

However, researchers have found numerous biomarkers associated with depression, but the statistical significance of each of these in isolation has not been strong enough to make a diagnosis but cross‐disciple research efforts are being made to combine these various results, measuring many of these genotypes, phenotypes and analytes to create tests that 1 day could potentially real‐world predictive power for MDD and greater success in antidepressant treatment outcomes. Encouragingly, advances are being made with the introduction of the FDA's “Biomarker Quantification Programme” in 2018 and the recent announcement of a glutamine+glutamate (Glx) pharmacodynamic biomarker for depression with a Letter of Support from the FDA for the first Glx‐targeted NMDA antidepressant (NRX‐101, combines D‐cycloserine, an NMDA receptor modulator; and lurasidone, a 5‐HT_2A_ receptor antagonist) entering Phase2b/3 studies. https://globenewswire.com/news-release/2018/05/07/1497324/0/en/NeuroRx-Receives-Special-Protocol-Agreement-SPA-and-Biomarker-Letter-of-Support-from-FDA-for-Pivotal-Studies-of-NRX-101-to-treat-Severe-Bipolar-Depression-in-Patients-with-Acute-Su.html)

## 16 CURRENT TREATMENT AND DRUG CLASSIFICATION

The majority of antidepressants in clinical use today act by enhancing the neurotransmission of monoamines, serotonin (5‐HT), NE, DA, or all 3, either directly or indirectly.^8^, ^33^, ^97^, ^98^, ^99^, ^100^, ^101^ This is done by either blocking reuptake via monoamine transporters or blocking the metabolism of monoamines by inhibiting the major catalytic degradation enzymes, monoamine oxidase, and catechol‐o‐methyltransferase (COMT).^97^, ^98^, ^100^, ^101^ Other modes of action include direct or indirect modulation of receptors or signal transduction mechanism. Antidepressants that modulate neurotransmission of monoamine uptake inhibition are divided into those that are nonselective (eg,TCAs, with dual action), SSRIs, and selective norepinephrine reuptake inhibitors (SNRls). An additional class of antidepressant is the polypharmic heterocyclics acting at both reuptake sites and receptors. Neuropeptide agents and transcription factors with potentially novel mechanisms of action, albeit possibility acting through the monoaminergic pathway are still under investigation,^8^, ^9^ (Figure 2). Of particular note, is the recent studies on “histone serotonylation” and the role of serotonin also regulating gene expression inside brain cells, which represent a dramatic divergence from the current dogma and mood disorders and the delayed response to SSRIs, according to the authors.^102^

### 16.1 The future: Alternative Non‐monoamine research strategies?

The monoamine hypothesis of depression has been the cornerstone of antidepressant treatment for 50 years, however, many questions remain unanswered as to the underlying pathophysiology of depressive/affective disorders and monoamines themselves are responsible for regulating depressives’ states. It is clear that the etiology of depression and bipolar disorder is still unknown. The fact remains the clinical response is delayed several weeks following administration of monoaminergic antidepressants suggesting that other mechanisms may well be involved in the efficacy of these agents. It has long been suggested that alterations in gene expression are contributing factors for the delayed clinical response, thereby resulting in changes in signal transduction mechanisms^33^, ^99^, ^102^ (see Figure 1).

Multiple mechanisms are likely to account for the clinical response in alleviating depression: (a) receptor downregulation; (b) other components of cellular signaling (eg, biased agonism) that are regulated by cyclic AMP, which are prominent transcription factors in the brain (phosphorylated cAMP response element protein, CREB); and (c) factors controlling cellular plasticity such as brain‐derived neurotrophic factor –,^103^ 18KDa translocator protein—TSPO,^104^, ^105^ Trace Amines—TAAR1.^8^, ^9^, ^106^, ^107^

One fascinating and potentially major step forward in our understanding the mechanism that contributes to the SSRI's treatment‐resistance observed in approximately 30% of MDD patients and may greatly aid patient stratification, was recently published by Fred Gage's group. This group studied serotonergic transmission in patient forebrain iPSC neurons in vitro and observed that nonremitter patient‐derived neurons displayed serotonin‐induced hyperactivity downstream of upregulated excitatory serotonergic receptors, in contrast to what is seen in healthy and remitter patient‐derived neurons.^108^ These findings suggest that postsynaptic forebrain hyperactivity downstream of SSRI treatment may play a role in SSRI resistance in MDD. These studies paint a complex and more nuanced picture of the serotonin hypothesis of depression and further highlight a role for serotoninergic dysfunction in the neuropathology of SSRI resistance in MDD that may lead to a further understanding endophenotypes and better treatment of MDD.

Aside from these recent advances, the number one challenge remains to develop novel antidepressants with greater efficacy and rapid action (see section on ketamine, Table 4). To this end, several pharmaceutical companies still continue to bet on the tried and tested monoamine approach and various augmentation strategies,^109^ the most recent monoamine entrants in the US depression market being duloxetine (2004), agomelatine (2009), vortioxetine (2013)**.** A comprehensive chart of compounds that fall within and outside the monoamine hypothesis currently in clinical development is presented in Figure 2 (taken from; https://clinicaltrials.gov/ and https://www.clinicaltrialsregister.eu, EUDRA.

## 17 CONCLUSION

Although we live amid a game‐changing revolution in neuroscience in the last decade, we are still incumbered with flawed biology, suboptimal regulatory models, clinical trial design, and protocols restricting progress. The dilemma faced by neuroscientists, regulatory authorities, the clinical community and patients, is where to place their faith in antidepressants, current diagnostic criteria, clinical trial design, and the rapid advances in the neuroscience. In recognition of the patient's needs, steps are being taken in the right direction with the emphasis on modernizing current regulatory pathways with the application of Master Protocols.^104^ Focusing on better metrics in target populations where there is an urgent need for developing or using alternative statistical methods more appropriate for data generated from innovative clinical practices along with technological advances. The management of change, however, that impacts progress often relate to policies that are the product of institutional cultures, which put political and financial incentives and bureaucratic procedures above patient's needs. The hope is that will change with the advent of novel robust biological and clinical data translating into patient benefit's and transforming and negating existing dogma for this debilitating and life‐threatening disease. There is an increasing need to take a holistic view of a personalized medicine and depression, for example looking at heart disease and depression together to understand how factors like epigenetics, traumatic experiences and the environment impact on both our physical and mental health.

Current advances in research show that changes in shared biological system are involved in many therapeutic fields. Thus, we need to stop thinking about mental and physical health in isolation and continue this example of bringing medical sciences together to create real change

Thus, the aim of this review has been to critically look at the failures of antidepressant drugs in clinical trials and address the issues and challenges for future development of novel antidepressants drugs. The need for multiple transformations and tangible advances in regulatory guidelines, drug labeling, diagnosis criteria, restrictive inclusion/exclusion, optimized clinical trials recruitment, approval of novel biomarkers/critical sample/biomarker analysis and advances in innovative data analytics to enable radical personalization are urgently required by all stake holders.

Therefore, as we emerge from this conceptual neurobiological revolution, the integrity and validity of epigenetic data, imaging brain neurocircuitry, molecular and structural insights, will become increasingly important in guiding optimal diagnosis, prediction of treatment responses in the discovery, and development of “better” antidepressants.

To quote Samuel Beckett: *“Try again. Fail again, Fail Better,” and to add to those wise words “To learn from our mistakes.”*
